# Radial Basis Function Neural Network with Localized Stochastic-Sensitive Autoencoder for Home-Based Activity Recognition

**DOI:** 10.3390/s20051479

**Published:** 2020-03-08

**Authors:** Wing W.Y. Ng, Shichao Xu, Ting Wang, Shuai Zhang, Chris Nugent

**Affiliations:** 1 Guangdong Provincial Key Lab of Computational Intelligence and Cyberspace Information, School of Computer Science and Engineering, South China University of Technology, Guangzhou 510006, China; wingng@ieee.org (W.W.Y.N.); sc_xu@foxmail.com (S.X.); 2 School of Computing, Ulster University, Shore Road, Newtownabbey, Co., Antrim BT37 0QB, Northern Ireland, UK; s.zhang@ulster.ac.uk (S.Z.); cd.nugent@ulster.ac.uk (C.N.)

**Keywords:** smart homes, activity recognition, localized generation error, autoencoder, stochastic sensitivity, radial basis function neural network

## Abstract

Over the past few years, the Internet of Things (IoT) has been greatly developed with one instance being smart home devices gradually entering into people’s lives. To maximize the impact of such deployments, home-based activity recognition is required to initially recognize behaviors within smart home environments and to use this information to provide better health and social care services. Activity recognition has the ability to recognize people’s activities from the information about their interaction with the environment collected by sensors embedded within the home. In this paper, binary data collected by anonymous binary sensors such as pressure sensors, contact sensors, passive infrared sensors etc. are used to recognize activities. A radial basis function neural network (RBFNN) with localized stochastic-sensitive autoencoder (LiSSA) method is proposed for the purposes of home-based activity recognition. An autoencoder (AE) is introduced to extract useful features from the binary sensor data by converting binary inputs into continuous inputs to extract increased levels of hidden information. The generalization capability of the proposed method is enhanced by minimizing both the training error and the stochastic sensitivity measure in an attempt to improve the ability of the classifier to tolerate uncertainties in the sensor data. Four binary home-based activity recognition datasets including OrdonezA, OrdonezB, Ulster, and activities of daily living data from van Kasteren (vanKasterenADL) are used to evaluate the effectiveness of the proposed method. Compared with well-known benchmarking approaches including support vector machine (SVM), multilayer perceptron neural network (MLPNN), random forest and an RBFNN-based method, the proposed method yielded the best performance with 98.35%, 86.26%, 96.31%, 92.31% accuracy on four datasets, respectively.

## 1. Introduction

Over the past few years, with the development of 5G and the advancement of Internet Protocol Version 6 (IPv6), the Internet of Things (IoT) paradigm has been greatly developed [1]. One particular instance of this is in the domain of smart homes where sensing devices have gradually entered into people’s lives. Collecting information through various sensors can identify an inhabitant’s activities and subsequently, following analysis, provide better services for them. To offer such a service requires a fundamental computational process of activity recognition where the data from the sensors embedded within the environment is processed. Activity recognition is a very critical and important process, which can be used to underpin a multitude of services such as personal health management [2], elderly care services [3], smart home services [4,5], to name but a few. However, it is a complicated process with many unsolved challenges such as multiple occupancy, interleaved activities, incomplete sensor data and differences in inter- and intra-inhabitant behaviors. As a result, due to the diversity of its data sources (smart home, smart wearable devices, mobile phones sensors and cameras), various solutions have been proposed to solve this problem [6]. 

Some research studies in the area of activity recognition have been based on image data collected by cameras [7,8,9,10]. Limited by computational complexity and privacy issues, this type of activity recognition is mainly used in the field of public safety and human-computer interaction. As an alternative to image-based data, some research has been based on data collected from wearable devices and smartphones [11,12,13]. In this paper, we focus on home-based activity recognition, which is mainly used in elderly care service or smart home service provision. Binary data were collected through binary sensors embedded in different locations/objects in the home environment, such as pressure sensors, contact sensors, passive infrared sensors etc. The outputs of the sensors are binary where the value is 1 with the sensor being activated and 0 otherwise. For example, a contact sensor is set on a door to provide binary information: the door is open or closed. In the home-based sensors system, sensors are embedded in the home environment, which are comfortable than the wearable device that should be worn every day. In addition, sensors only obtain binary information, like ON/OFF, OPEN/CLOSE etc., which would not cause privacy issues that could be raised when, for example, using a camera. Home-based activity recognition approaches use sensors to capture various interactive information generated by inhabitants within their home environment, and subsequently extract key information to identify user activities [14]. These methods recognize the activities of daily living, like watching TV, taking a shower, using the toilet and sleeping, rather than simple actions such as raising hands or sitting down as may be the case in image analysis. 

There are some challenges associated with the home-based activity recognition. Firstly, by contrast with the continuous data collected from cameras or the triaxial acceleration sensor in smart wearable devices, home-based sensors data are mostly binary, which carry less information. Therefore, the collected sensors data may not be enough to provide a full representation of the activities [14]. Secondly, sensors data may be unreliable due to the transmission error or hardware problems [15]. Besides that, with different living environments, various sensors systems with different sensors are proposed to collect data [16]. This requires methods to have high generalization ability to cope with the recognition tasks under different environments and using different sensor systems. In an effort to address these challenges, methods should be applicable to different environments, in addition to addressing the unreliability of the low-level sensor information. In our opinion, the nature of home-based activity recognition is to extract key information that can be used to determine a user’s activities from a range of multiple heterogeneous sensors. In this work, a radial basis function neural network (RBFNN) with localized stochastic-sensitive autoencoder (LiSSA) is proposed to solve this problem. Binary data collected by multiple sensors embedded in the home environment like pressure sensors, contact sensors and passive infrared sensors are used as inputs in our method, and one sensor corresponds to one input dimension. Firstly, an autoencoder (AE) is used to encode data obtained from multiple sensors, in order to extract features that may be important. In this step, AE converts binary inputs into continuous inputs, which help to extract additional hidden information. In addition, the generalization ability of the AE is also considered. The training of the AE is based on the localized generalization error (LGE) [17], in order to improve the model’s ability of facing instances of unreliable sensor data. The LGE model has been widely applied in different applications, for instance, neural network architecture selection [17], sample selection [18] and multilayer perceptron neural network (MLPNN) training [19]. Combining the AE with the RBFNN, a LiSSA-RBF model is conceived. LiSSA-RBF is compared with other five benchmarking approaches, and yields the best performance. The contributions in this study are as follows:

- We proposed a novel hybrid artificial neural network (ANN) model LiSSA-RBF for the home-based activity recognition problem.
- The proposed method takes the stochastic sensitivity measure into consideration, which is used to quantitatively measure the network output fluctuations relative to the small disturbance of the network input to tolerate the uncertainty of low-level sensor data. By using LiSSA, feature extraction and the unreliable data are under consideration at the same time, and provide more reliable and effective features for activity recognition.
- We compared the performance of various methods on four different activity-recognition datasets from different environments. The high performance achieved by the proposed method shows its effectiveness and could be applicable to different environments.

Related work of home-based activity recognition is discussed in Section 2. The proposed method is presented in Section 3. Section 4 introduces four datasets we use and analyzes the performance of different methods on these datasets. Conclusions and future work are presented in Section 5.

## 2. Related Work

Activity recognition is a very critical and important process, which can be used to underpin a multitude of services [16]. Many approaches explore activity recognition from various perspectives, including the video-based methods [7,8,9,10] and wearable device-based methods [11,12,13,20]. However, these methods are limited by privacy issues or comfort. Home-based activity recognition has recently attracted considerable research interest. Compared with video-based and wearable device-based approaches, home-based approaches make full use of sensing devices in smart homes, which are non-invasive to people without affecting people normal lives. Many sensor systems are used in the home environment for activity recognition [16]. Barger et al. [21] constructed a system of distributed passive infrared sensors to collect a person’s activities data. In [22], eight passive infrared sensors are installed in the ceiling of rooms to collect activities data. The activities are classified by checking the number of sensors activated and recording the time interval for which they remain activated. Lee et al. [23] described a system which is formed by an array of passive infrared sensors and locates a resident with a reasonable accuracy by combining the overlapping detection areas of adjacent sensors. More different sensor systems for activity detection are described in [16,24,25,26]. 

In addition, many machine-learning methods on smart homes have been proposed [27], such as process mining [28], active learning and dynamic K-means [29]. Van Kasteren et al. recorded 25 days’ activities data in the home of a 26-year-old male by using a wireless sensor network [30]. 14 binary sensors were used to collect 7 types of activity, including taking a shower and preparing dinner. These real-world data were used to evaluate the effects of different probabilistic models, including the hidden Markov model (HMM), conditional random fields (CRF), hidden semi-Markov models (HSMM) and semi-Markov conditional random fields (SMCRF). In their experiments, HSMM, CRF and SMCRF all significantly outperformed HMM. Besides that, SMCRF, despite their greater expressive power, did not result in significant performance increase. To improve the effectiveness of a single activity recognition model, a hybrid method combining HMM and support vector machine (SVM) is proposed in [31]. The hybrid HMM/SVM method performed better than single models on all five datasets, and the differences were significant on three datasets. This demonstrated how hybrid schemes can be effectively employed for activity recognition.

Besides probabilistic models, the ANN, which has the ability to implicitly detect complex non-linear relationships between data and their classifications, is also widely used in home-based activity recognition [32,33,34]. Gochoo et al. proposed a deep convolutional neural network (DCNN) classification approach to detect four basic activity classes [35]. They converted a sequence of passive infrared sensor logs into an activity image, and then implemented a DCNN classifier to detect activities. This approach made full use of the advantages of deep networks in image processing, and produced an accuracy of 99.36% for the four activities recognition. Although this approach performed well on the dataset with only four types of activity, more complex situations need to be investigated.

Chiang et al. [36] explored the application of transfer learning in activity recognition. Most current machine learning methods require a large amount of label data for each house, and will be powerless with the lack of labeled data. Authors proposed a framework for knowledge transfer with SVM and RBF to solve this problem. They tried to reuse learned knowledge from an existing environment (a house with enough labeled data) into another one (lack of labeled data). The results showed that in most cases, the accuracy is more than 70% when using knowledge transfer.

An ensemble of long short-term memory (LSTM) with fuzzy temporal windows method was proposed to solve real-time recognition of interleaved activities in [37]. The authors proposed a representation of binary-sensor activations based on multiple fuzzy temporal windows and then trained an ensemble of LSTM [38] classifiers. This method presented an F1-score in real-time close to 75%. Furthermore, Singh et al. [39] compared the performance of CNN, LSTM with four other methods including Naïve Bayes, HMM, HSMM and CRF on three home-based activity recognition datasets. Experiment showed that these two ANN methods outperformed the four other methods, and the LSTM yielded the best results. 

Aiming to exploit deep-learning techniques to learn high-level features from the binary sensor data, a stacked denoising autoencoder (SDAE) was implemented in [40]. The SDAE was used to learn the discriminant latent patterns inherent in the low-level features. In this study, SDAE and activity recognition were unified in a single framework. The experiments on three activity-recognition datasets showed that this method achieve better performance than HMM and Naïve Bayes methods, however, it performed similarly to SVM.

These methods all face the problem of not being able to fully accommodate instances of unreliable sensor data [14,15]. Hong et al. [15] proposed a framework to handle this situation. The unreliable sensor data were accommodated by the use of a series of information-handling techniques, including the Dempster–Shafer theory of evidence and the equally weighted sum operator. 

RBFNN is a classic type of ANN, which has efficient training speed and the capability of approximating a function with any precision rate given enough hidden neurons [14]. Therefore, RBFNN is widely used in various fields [41,42,43]. Zhang et al. [14] focused on strengthening the capabilities of models, and proposed an RBFNN based on LGE to address the tolerance issues surrounding low-levels of uncertain sensor data. This method focused on improving the generalization ability of the model by minimizing LGE. Compared to other activity recognition methods including SDAE, SVM and the original RBFNN, this method yielded the best performance on all four datasets. This revealed the importance of model generalization abilities in activity recognition.

## 3. Methodology

In this Section, the LiSSA-RBF method is proposed to solve the activity-recognition problem. This method simultaneously considers two challenges in home-based activity recognition, low-level information data and unreliable data. An AE is used to extract potential relevant information from the binary sensor data. Besides that, the LGE model in [14] is used to train the AE, in order to improve the AE ability of facing instances of unreliable sensor data, extract more reliable features for activity recognition. The AE is introduced in Section 3.1, and the AE trained by the LGE model is introduced in Section 3.2. Finally, the proposed LiSSA-RBF method is introduced in Section 3.3. 

### 3.1. Autoencoder (AE)

An AE is a type of ANN which is used for representation learning. A single hidden layer AE can be expressed as follows:

(1) $$Y=\varphi_{2}\left(\varphi_{1}\left(XW+b_{1}\right)V+b_{2}\right),$$

where *X* is the input vector with *d* dimension, represents the data that need to be encoded. *Y* is the output vector with *d* dimension, represents the decoded data. ***W***, ***V*** are the weight matrices from input-layer and hidden-layer, and $\varphi_{1}$,$\varphi_{2}$,$b_{1}$,$b_{2}$ are the activation functions, the bias of hidden-layer and output-layer, respectively.

An AE can be simply abstracted into two parts: encoding and decoding. Let

(2) $$C=\varphi_{1}\left(XW+b_{1}\right),$$

we have,

(3) $$Y=\varphi_{2}\left(CV+b_{2}\right)$$

**Figure 1:**
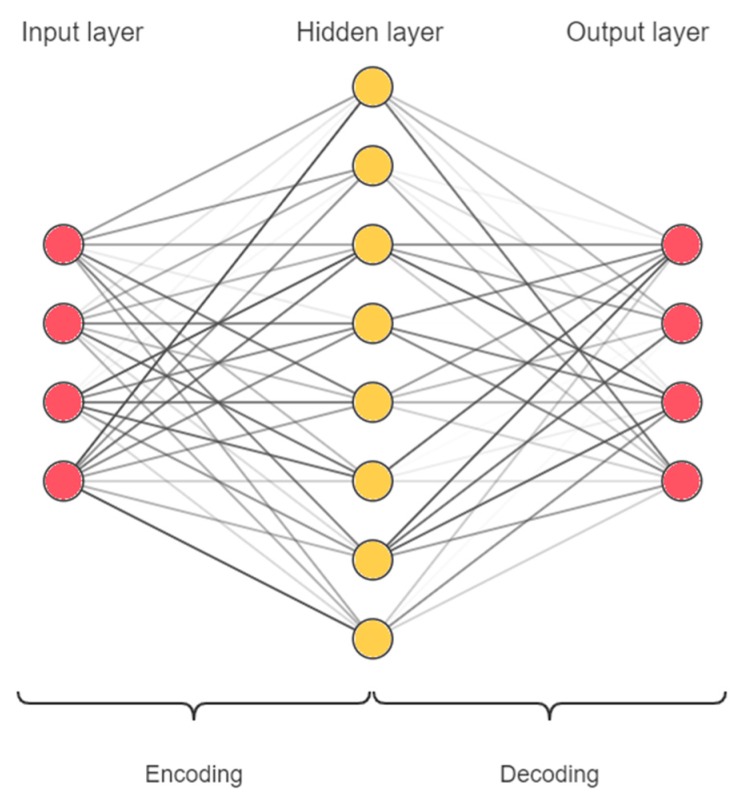
The structure of an autoencoder (AE), including input layer, hidden layer and output layer.

As presented in Figure 1, the AE obtains the reconstructed input *C* by the encoding process Equation (2), and then decodes it to restore the original input Equation (3). The goal is to ensure that the encoded data *C* retains the information from the original input and can be decoded into the original input accordingly. Therefore, an AE can be trained by the back-propagation algorithm, with the goal of minimizing the mean square error between the output and input, that is:

(4) $${\mathrm{argmin}}_{W,V,b_{1},b_{2}}\frac{1}{N}\sum_{i=1}^{N}\|Y^{\left(i\right)}-X^{\left(i\right)}{\|}^{2},$$

where $X^{\left(i\right)}$ denotes the *i*-th sample, ‖·‖ means the 2-norm of vector, and *N* is the number of samples.

Depending on different activation functions and different numbers of hidden layer units, the AE will achieve different effects. For example, when the activation function is a linear function and the number of hidden layer units is less than the input dimension *d*, the AE will perform a linear dimensionality reduction on the input [44]. In this paper, without losing generality, we use the non-linear function sigmoid function f(x)=1/(1+exp(−x)) as the activation function and set the number of hidden layer units greater than the input dimension. This structure can perform non-linear dimensionality enhancement on the input, which is helpful to learn hidden and useful features and to extract the implicit relationships between the input features [44].

### 3.2. Localized Stochastic-Sensitive Autoencoder (LiSSA)

The traditional AE, as mentioned in the previous Section, is trained with the goal of minimizing the mean square error of the training data, that is:

(5) $$R=\frac{1}{N}\overset{N}{\underset{i=1}{\sum }}\|Y^{\left(i\right)}-X^{\left(i\right)}{\|}^{2}$$

These cannot evaluate the generalization ability of the AE on the reconstruction input for those unseen samples. Therefore, we use a training method based on LGE to train the AE, referred to as LiSSA [45]. The goal of the LGE-based training method is to achieve a low generalization error for future unseen samples [46]. For machine-learning tasks, those unseen samples are usually near the training samples and do not exceed a distance *Q*, otherwise this training sample is not representative of the given problem. The range where unseen samples are located is called the *Q*-neighborhood:

(6) $$S_{Q}\left(X\right)=\left\{X^{*}|X^{*}=X\pm \Delta X\right\},\;\left|\Delta X\right|\in {\left[-Q,Q\right]}^{d},$$

where ΔX denotes the perturbations to the sample X.

Then, LGE is defined as follows:

(7) $$LGE\left(X,Q\right)=\int_{S_{Q}\left(X\right)}\|Y-X^{*}{\|}^{2}p\left(X^{*}\right)dX^{*},$$

where Y is the AE output in Equation (1), and p(X) denotes the probability density function of X in $S_{Q}\left(X\right).$

For all training samples, the LGE would be:

(8) $$LGE\left(Q\right)=\frac{1}{N}\overset{N}{\underset{i=1}{\sum }}\int_{S_{Q}\left(X^{\left(i\right)}\right)}\|Y-X^{*}{\|}^{2}p\left(X^{*}\right)dX^{*}$$

Assume that the unseen samples within the *Q*-neighborhood follow a normal distribution and apply Hoeffdings inequality with a probability of 1−η to Equation (8), we have:

(9) $$LGE\left(Q\right)\leq {\left(\sqrt{R}+\sqrt{E_{S_{Q}}\left({\left(\Delta Y\right)}^{2}\right)}+A\right)}^{2}+B\cdot \sqrt{\frac{ln\left(\eta \right)}{-2N}}=LGE^{*}\left(Q\right),$$

where ΔY=g(X)−g(X+ΔX), and g(X) denotes the mapping from X to Y in Equation (1), A and B denote the maximum output difference and the maximum possible value of the mean square error (MSE), respectively. The $E_{S_{Q}}\left({\left(\Delta Y\right)}^{2}\right)$, named as the stochastic sensitivity measure (STSM), can be calculated by a quasi-Monte Carlo-based method. Specifically, ΔX is generated via an *n*-dimensional Halton sequence [47] with each coordinate ranging from [−Q,Q] and 50 Halton points are used in the calculation:

(10) $$E_{S_{Q}}\left({\left(\Delta Y\right)}^{2}\right)\approx \frac{1}{N}\overset{N}{\underset{i=1}{\sum }}\overset{50}{\underset{j=1}{\sum }}\|g\left(X^{\left(i\right)}\right)-g\left(X^{\left(i\right)}+\Delta X_{j}\right){\|}^{2}$$

Therefore, under the same assumption, for the same training sample, minimizing LGE can be transformed into minimizing $\sqrt{R}+\sqrt{E_{S_{Q}}\left({\left(\Delta Y\right)}^{2}\right)}$.

### 3.3. LiSSA-Radial Basis Function (RBF) Network

In the proposed approach, a RBFNN is used for the purposes of activity recognition due to its effective training speed and the ability to approximate the function with any accuracy given sufficient numbers of hidden neurons. The experiment in [14] demonstrated the effectiveness of the RBFNN and RBFNN-based method in activity recognition. The RBFNN is a three-layer ANN. The activation function of the output layer is a linear function f(X)=X, and the activation function of the hidden layer is shown as follows:

(11) $$\varphi \left(X\right)=e^{\frac{\|X-u{\|}^{2}}{-2\sigma^{2}}},$$

where u, σ denote the center and variance of the radial basis function.

**Figure 2:**
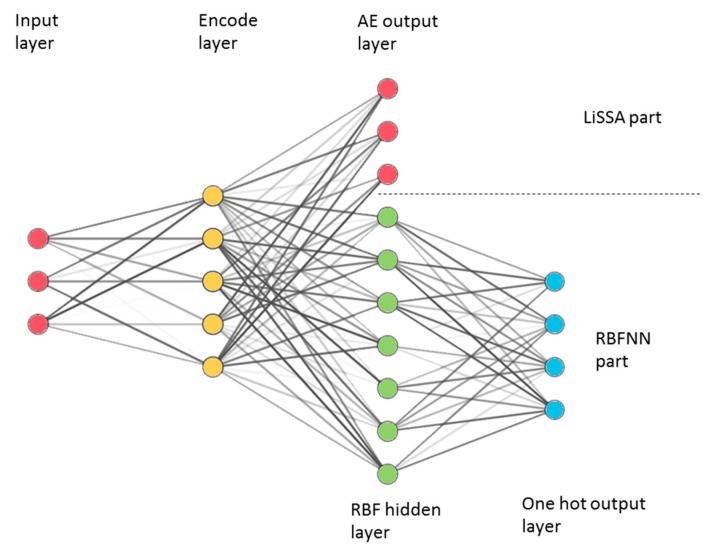
Localized stochastic-sensitive autoencoder-radial basis function (LiSSA-RBF) network structure, consist of the LiSSA part (input layer, encode layer and AE output layer) and the radial basis function neural network (RBFNN) part (Input layer, encode layer, RBF layer and one hot output layer).

The center and variance are calculated by a cluster method *k*-means algorithm on all training samples, and each hidden layer unit corresponds to a cluster center. Connecting the RBF network with the LiSSA proposed in Section 3.2, the final network structure is obtained (Figure 2). The process of the proposed method is divided into two steps. Step one is to train LiSSA according to the goals in Section 3.2 and extract features from the input. Step two is to learn the parameters of the RBF layer and the weights between the RBF layer and the one output layer. In step two, the weight of the encode layer trained in step one will not be adjusted. This structure and training steps introduce LiSSA to perform feature extraction for RBFNN, which may strengthen RBFNN capability. 

The number of RBF layer units *r*, which is also the number of cluster centers of the k-means algorithm, is determined by a greedy algorithm based on minimizing the training MSE presented in Algorithm 1.
**Algorithm 1.** Greedy search algorithm for Localized stochastic-sensitive autoencoder-radial basis function (LiSSA-RBF) network.   **Input:**
     Number of activity types: *p*     Number of training samples: *N*     Training dataset: (X,Y)   **Output:**
     The final LiSSA-RBF network   1. Set the number of radial basis function (RBF) layer units *r* equal to the number of activity types *p*;   2. Construct and train a LiSSA-RBF network with *r* RBF layer units;   3. Compute the training mean square error (MSE) of the trained LiSSA-RBF network;   4. If r<N/2, let r=r+1 and go to step 2, otherwise go to step 5.   5. The LiSSA-RBF network with minimum training MSE is picked as the final network structure.

The flow of using LiSSA-RBF to recognize activities is as follows. Firstly, all training binary sensors data are sent into LiSSA part to learn the mapping to the high-dimensional features. Secondly, the LiSSA part is used to map binary sensors data to high-dimensional space as input, and use one-hot encoded activities types as output to train the RBF part with a greedy algorithm shown in Algorithm 1. So far, the training of LiSSA-RBF has been completed. Finally, the activity data collected by sensors is sent into LiSSA-RBF, after the feature extraction by the LiSSA part and the classification by RBF part, and the recognition result will be output.

## 4. Experiment and Evaluation

This Section analyzes the effects of the proposed method experimentally. Section 4.1 describes the data used in the experiment. Section 4.2 describes the evaluation indicators used. Section 4.3 compares the effects of the proposed method with other methods and analyzes the influence of the number of hidden layer units in LiSSA on the proposed method.

### 4.1. Datasets

Four binary datasets including OrdonezA, OrdonezB [31], Ulster [14], and activities of daily living data from van Kasteren (vanKasterenADL) [30] have been used to evaluate the effectiveness of the proposed method. Various sensors were used to collect data, including pressure sensors, contact sensors and passive infrared sensors. The information collected by these sensors has one thing in common: they only produce binary outputs. 

Datasets OrdonezA and OrdonezB are two activity datasets belonging to two persons in different houses, collected by wireless sensor networks with 12 sensors and 10 sensors in total, respectively. Five types of sensors are used in OrdonezA and OrdonezB, including passive infrared sensor, magnetic sensor, flush sensor, pressure sensor and electric switch. Sensors are distributed in the kitchen, living room, toilet and bedroom. All data were manually labeled. OrdonezA includes 14 days’ data, consisting of 242 activity instances and 9 activity types. OrdonezB has 21 days’ data in total, with 482 activity instances and 10 activity types.

The vanKasterenADL dataset includes 7 activity types and 242 activity instances, which were performed by a 26-year-old male over a period of 25 days. 14 sensors are installed in the home consist as a wireless sensor network to collect binary data, including reed switches, mercury contacts, passive infrared sensors and float sensors. One was in the toilet, nine were in the kitchen and four were on the hall-toilet door, hall-bathroom door, hall-bedroom door and front door, respectively. 

The Ulster dataset has been generated by a simulation tool referred to as the Intelligent Environment Simulation [48]. This tool provides a variety of sensors including passive infrared sensors, pressure sensors, and contact sensors for use. In the Ulster dataset, 21 virtual sensors were used to collect data, including 11 activity types and 308 activity instances. Table 1 summarizes the number of activity instances, activity types and sensors from each of the datasets, and Table 2 presents the specific activity types in four datasets.

### 4.2. Evaluation Indicators

Two indicators have been used to evaluate the effectiveness of the different methods being considered. The first is accuracy, which statistically identifies the correct and incorrect samples and calculates the accuracy of the recognition process. Nevertheless, when identifying multiple activities, the samples in the dataset are usually unbalanced. In the highly unbalanced situation, a simple classifier that predicts each instance as the majority class can achieve a high accuracy, which is not expected. To avoid this situation, the F1_score indicator is used as the second indicator, which takes precision and recall into consideration.

Table 3 is a multi-class confusion matrix, the number 1, 2, 3 denote three different activities. Rows represent the number of samples inferred as corresponding activities, and the columns represent the number of samples that actually belong to the corresponding activities.

For each activity, its own F1_score can be calculated as follows:

(12) $$F1\_{\mathrm{score}}_{i}=\frac{2\cdot precision_{i}\cdot recall_{i}}{precision_{i}+recall_{i}},$$

where:

(13) $$precision_{i}=\frac{TP_{i}}{SI_{i}}$$

(14) $$recall_{i}=\frac{TP_{i}}{ST_{i}}$$

After obtaining the F1_score of each activity, the average F1_score is calculated weighted by support (the number of true instances for each activity):

(15) $$F1\_\mathrm{score}=\sum_{i=1}^{p}ST_{i}\cdot F1\_{\mathrm{score}}_{i},$$

where *p* denotes the number of activities.

In order to obtain a more reliable evaluation, 10-fold cross-validation experiments were performed five times.

### 4.3. Experiment Results and Discussion

In this Section, the proposed method (LiSSA-RBF) is compared with other well-established machine-learning methods, including random forest, MLPNN, and SVM. Some of them performed well in [14,40].The proposed algorithm is also compared with the LGE-based RBF (RBF_LGEM) method [14] and SVM with LiSSA (LiSSA-SVM) to prove the effectiveness of the proposed algorithm structure. MLPNN used in the experiment is a standard three-layer neural network, with the sigmoidal activation function for the hidden layer. Since the activity recognition problem is a multi-classification problem, softmax activation function is used in the output layer for MLPNN. The methods of random forest, MLPNN, SVM and RBF_LGEM take the raw sensor features as input, while methods LiSSA-SVM and LiSSA-RBF use LiSSA as feature extraction first. To control variables, LiSSA-SVM and SVM use the same linear function as their kernel function, and the AE in LiSSA-SVM and LiSSA-RBF have the same structure and parameters. All methods are implemented and calculated by python 3.6, on a computer with Intel Core i5–7500 central processing unit (CPU) and 8 GB RAM.

Table 4 and Table 5 are the average accuracy and F1_score values of different methods on four datasets, respectively. It can be viewed that on all datasets, LiSSA-RBF yields the best performance on accuracy and F1_score. Compared with the RBF_LGEM method, LiSSA-RBF has been improved by 0.83%, 0.37%, 1.05%, 1.79% on accuracy, and 0.84%, 0.38%, 0.94%, 2.28% on F1_score. The t-test shows that on dataset OrdonezA, Ulster and vanKasterenADL, LiSSA-RBF is significantly better than RBF_LGEM with a significance level of 0.05. LiSSA-RBF and RBF_LGEM both use RBFNN as classifier to recognize different activities, and also use LGE to improve model’s ability of facing instances of unreliable sensor data. The difference is that the proposed method introduces LiSSA to extract useful features, which greatly improves the model’s ability. This demonstrates the importance of using LiSSA to assist RBF in feature extraction. On the other hand, LiSSA-SVM performs better than SVM on the OrdonezA and Ulster datasets, while it performs worse than SVM on OrdonezB and vanKasterenADL. Seems that LiSSA do not work well with SVM. This is because the SVM algorithm has used the kernel function to perform the feature extraction operation, making LiSSA unable to achieve a further stable improvement for SVM. For this analysis, the LiSSA-RBF structure is better than the LiSSA-SVM structure, because LiSSA-RBF takes full advantages of AE and RBFNN, while LiSSA-SVM does not. The experimental results also show that LiSSA-RBF performs better than LiSSA-SVM on all four datasets. In comparison with other methods, LiSSA-RBF always yields the best results in all four datasets, which demonstrates the high generalization ability of the proposed method.

As mentioned above, the proposed method does not significantly outperform RBF_LGEM on the dataset OrdonezB. Besides that, most methods do not perform well on dataset OrdonezB in our experiment. Therefore, we analyze the performance of the proposed method on dataset OrdonezB. Table 6 and Table 7 show the confusion matrix of proposed method and RBF_LGEM on dataset OrdonezB, respectively. Focusing on the incorrectly recognized samples, we find that misrecognition occurred frequently among activity 1, 2, 4 and 8. These four activities are breakfast, dinner, lunch and snack, which are similar to each other and actually represent a same behavior, eating. We checked the sensor data for these activities, and found that some of the sensor data were exactly the same. This is understandable because the interactions of these activities with the environment are almost the same. However, in this condition, the proposed method or other data-driven methods do not work well, which would reduce the accuracy and F1_score of recognition. To solve this problem, introducing more information is necessary. For example, using more sensors to collect more information, or considering the time when the activity occurred and the previous activity type.

**Figure 3:**
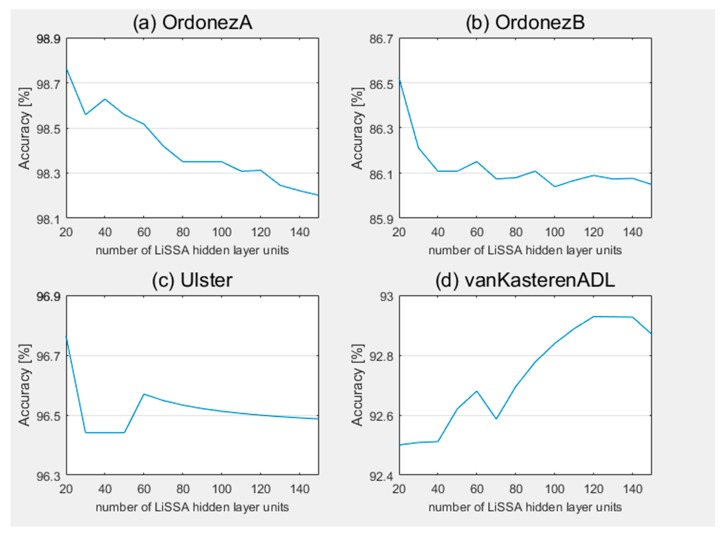
Accuracy of proposed method with different number of LiSSA hidden layer units on datasets (**a**) OrdonezA, (**b**) OrdonezB, (**c**) Ulster, and (**d**) vanKasterenADL.

**Figure 4:**
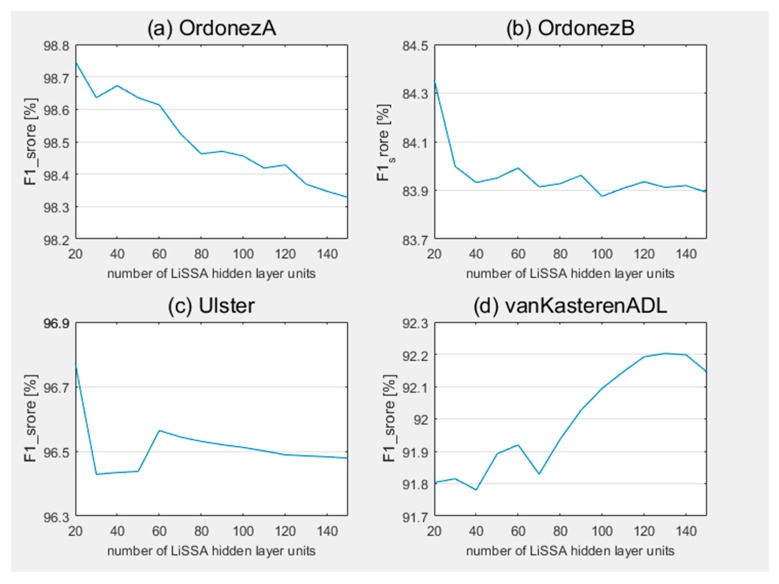
F1_score of proposed method with different number of LiSSA hidden layer units on datasets (**a**) OrdonezA, (**b**) OrdonezB, (**c**) Ulster, and (**d**) vanKasterenADL.

Secondly, we analyzed the effect of the number of LiSSA hidden layer units on the proposed method. Figure 3 and Figure 4 present the change of accuracy and F1_score with different number of LiSSA hidden layer units on the proposed method of the four datasets, respectively. For all datasets, the change ranges are all about 0.5%. On the vanKasterenADL dataset, with the increase of the number of hidden layer units, the accuracy and F1_score gradually improved in the overall trend, and achieved the best result on 140 hidden layer units. As described in Section 4.1, sensors are installed in the kitchen and toilet only. That means some activities in other rooms, like sleeping, cannot be directly perceived by sensors. These activities need to be judged by the potential relevant information in sensors set in other rooms. Therefore, more hidden layer units are required to provide sufficient hidden features. On the other hand, the proposed method achieved the best results at 20 hidden layer units on datasets OrdonezA, OrdonezB and Ulster, and the performance gradually decrease as the number of units increase in the overall trend. This shows that on these three datasets, 20 hidden layer units are sufficient to extract representative features for activity recognition, and higher numbers may introduce more noise, leading to decreased recognition performance.

In summary, it has been found that the number of hidden layer units will affect the performance of the proposed method to a certain extent. The optimal number of hidden layer units is determined by the nature of a dataset. For more complicated situations, more hidden layer units are required, otherwise only a small number of hidden layer units are required.

## 5. Conclusions

In this paper, an RBFNN with a localized stochastic-sensitive autoencoder is proposed to solve the sensor-based activity recognition problem within smart home environments. Based on an RBFNN activity-recognition model, the proposed method uses LiSSA for feature extraction to obtain improved recognition results. In addition, LiSSA is trained base on LGEM, which take both training errors and STSM into account. This approach solves the problems caused by the uncertainty of low-level sensor readings and improves the robustness of feature extraction. To evaluate the effectiveness of the proposed method, four datasets and two evaluation indicators have been used. Compared with other benchmarking methods, the proposed method performs optimally on all four datasets, revealing the effectiveness of the proposed algorithm and structure. In addition, we also analyzed the effect of the number of LiSSA hidden layer units on the proposed method. Experiments demonstrated that on three datasets, 20 hidden layer units are enough, and more units would introduce noise. For the remaining dataset, due to the unevenness of its sensors’ distribution, more hidden layer units are needed to obtain better results.

In the field of machine learning, classifier ensembles are the common techniques to improve model performance, which integrate the results from multiple models instead of relying on a single model. For example, the random forest method in Section 4.3 is an ensemble method combining multiple decision tree models. An element of the future works will focus on the ensemble method based on LiSSA feature extraction to further improve the effectiveness of the model. We will comprehensively consider the ensemble on the LiSSA part and on RBFNN part, and may also fuse other machine-learning models like decision trees. Our target is to build a model with high generalization ability to cope with reorganization tasks under different environments. At the same time, we will also collect more data from different sensor systems to test the effectiveness of the model. On the other hand, the proposed method uses activity instances independently, and obtains limited information. We do not make use of the information in the time dimension. For example, by introducing time information, it would become easier to distinguish between breakfast, lunch and dinner activities. Besides that, some activities are also related in sequence. It would be interesting to explore the LiSSA-RBF method, and enable it to take the sequence of activity types and the activity interval into account. To achieve this target, using the recurrent neural network framework based on LiSS-RBF may be a viable way forward. 

## Figures and Tables

**Table 1:** Simple description of four datasets.

Dataset	#Activity Instances	#Activity Types	#Sensors
OrdonezA [31]	242	9	12
OrdonezB [31]	482	10	10
UIster [14]	308	11	21
vanKasterenADL [30]	242	7	14

**Table 2:** Specific activity types in four datasets.

Dataset	Specific Activity Types
OrdonezA	leaving, toileting, showering, sleeping, breakfast, lunch, dinner, snack, TV, grooming
OrdonezB	leaving, toileting, showering, sleeping, breakfast, lunch, snack, TV, grooming
UIster	leave house, use toilet, take shower, go to bed, prepare breakfast, prepare dinner, get drink
vanKasterenADL	go to bed, use toilet, watch TV, prepare breakfast, take shower, leave house, get cold drink, get hot drink, prepare dinner, get dressed, use telephone

**Table 3:** Multi-classes confusion matrix, the number 1, 2 and 3 denote three different activities.

	True	1	2	3	Sum
Inferred					
1		*TP_1_*	*F_1,2_*	*F_1,3_*	*SI_1_*
2		*F_2,1_*	*TP_2_*	*F_2,3_*	*SI_2_*
3		*F_3,1_*	*F_3,2_*	*TP_3_*	*SI_3_*
Sum		*ST_1_*	*ST_2_*	*ST_3_*	*Total*

**Table 4:** Accuracy of different methods on four datasets. Results are obtained from 10-fold cross validations conducted five times.

Methods	OrdonezA [%]	OrdonezB [%]	Ulster [%]	vanKasterenADL [%]
Random Forest	97.77 ± 0.56	85.94 ± 0.24	95.01 ± 0.34*	89.40 ± 0.68*
MLPNN	78.38 ± 0.99*	84.48 ± 0.33*	95.85 ± 0.13*	78.95 ± 1.73*
RBF_LGEM	97.52 ± 0.26*	85.89 ± 0.25	95.26 ± 0.16*	90.50 ± 1.16*
SVM	97.02 ± 0.31*	85.56 ± 0.38*	95.40 ± 0.13*	91.80 ± 0.38
LiSSA-SVM	97.36 ± 0.21*	84.43 ± 0.30*	96.11 ± 0.01	90.40 ± 0.72*
LiSSA-RBF	98.35 ± 0.27	86.26 ± 0.42	96.31 ± 0.32	92.31 ± 0.85

The symbol ‘*’ indicates that the proposed method LiSSA-RBF is significantly better than this method with a significance level of 0.05.

**Table 5:** F1_score of different methods on four datasets. Results are obtained from 10-fold cross validations conducted five times.

Methods	OrdonezA [%]	OrdonezB [%]	Ulster [%]	vanKasterenADL [%]
Random Forest	97.82 ± 0.50*	84.80 ± 0.39	94.98 ± 0.42*	89.45 ± 0.72*
MLPNN	71.87 ± 1.18*	82.30 ± 0.46*	95.71 ± 0.32	72.96 ± 1.90*
RBF_LGEM	97.57 ± 0.37*	84.83 ± 0.32	95.25 ± 0.27*	90.32 ± 1.18*
SVM	97.03 ± 0.31*	84.31 ± 0.56*	95.37 ± 0.28*	91.78 ± 0.50
LiSSA-SVM	97.37 ± 0.20*	83.19 ± 0.27*	96.06 ± 0.24	90.75 ± 0.59*
LiSSA-RBF	98.41 ± 0.21	85.11 ± 0.47	96.19 ± 0.51	92.60 ± 0.66

The symbol ‘*’ indicates that the proposed method LiSSA-RBF is significantly better than this method with a significance level of 0.05.

**Table 6:** Confusion matrix of LiSSA-RBF on dataset OrdonezB. The activity ID from 1 to 10 represents breakfast, dinner, grooming, leaving, lunch, showering, sleeping, snack, watching TV, toileting, respectively. Rows represent the inferred activity and columns represent the actual activity.

Activity ID	1	2	3	**4**	5	6	7	8	9	10
**1**	8	0	0	0	3	0	0	4	0	0
**2**	0	3	0	0	0	0	0	0	0	0
**3**	0	0	91	0	0	0	0	0	0	1
**4**	0	0	0	38	0	0	1	0	1	0
**5**	3	0	0	0	3	0	0	3	0	0
**6**	0	0	0	0	0	11	0	0	0	0
**7**	0	0	1	0	0	0	27	0	0	0
**8**	10	8	0	0	5	0	0	32	0	0
**9**	1	0	8	0	2	0	1	8	114	2
**10**	0	0	4	0	0	0	0	0	0	89
**Accuracy [%]**	36%	27%	88%	100%	23%	100%	93%	68%	99%	97%

**Table 7:** Confusion matrix of the localized generalization error-based RBF method (RBF_LGEM) on dataset OrdonezB. The activity ID from 1 to 10 represents breakfast, dinner, grooming, leaving, lunch, showering, sleeping, snack, watching TV, toileting, respectively. Rows represent the inferred activity and columns represent the actual activity.

Activity ID	1	2	3	4	5	6	7	8	9	10
**1**	8	2	0	0	0	0	0	6	0	0
**2**	0	0	0	0	0	0	0	0	0	0
**3**	0	0	91	0	0	0	0	0	2	1
**4**	0	0	0	38	0	0	1	0	1	0
**5**	3	1	0	0	4	0	0	3	0	0
**6**	0	0	0	0	0	11	0	0	0	0
**7**	0	0	1	0	0	0	27	0	0	0
**8**	10	8	0	0	7	0	0	30	0	0
**9**	1	0	8	0	2	0	1	8	112	2
**10**	0	0	4	0	0	0	0	0	0	89
**Accuracy [%]**	36%	0%	88%	100%	31%	100%	93%	64%	97%	97%
